# Measuring success of targeted screening and prevention for hemoglobinopathies

**DOI:** 10.3389/fpubh.2025.1587738

**Published:** 2025-09-01

**Authors:** Rajat Kumar Agarwal, Sundar Periyavan, Deepa Trivedi, Vaibhav Shah, Mohana Reddy, George Mani, Amit Sedai, Kumari Ankita, Lawrence Faulkner

**Affiliations:** ^1^Sankalp India Foundation, Bangalore, India; ^2^Jagriti Innohealth Platforms Pvt. Ltd., Bangalore, India; ^3^Cure2children Foundation, Firenze, Italy

**Keywords:** screening, prevention and control, hemoglobinopathies, program evaluation, thalassemia and sickle cell disease

## Abstract

Targeted screening for hemoglobinopathies is crucial for preventing affected births and mitigating the psychological and financial burdens on families. This study proposes two novel indicators—Screening index and prevention index—to evaluate the effectiveness of hemoglobinopathy prevention programs. The Screening index measures the proportion of at-risk women who are successfully screened and informed, while the Prevention Index assesses the number of women enrolled per birth prevented, reflecting the program’s efficiency in reducing affected births. These indicators account for critical factors such as counselor quality, testing accuracy, timeliness, accessibility, and demographic influences, which impact program success. We also address the limitations of traditional measures and emphasize the need for normalized indicators to adjust for variations in carrier rates across different populations. This approach enhances program monitoring, informs resource allocation, and guides improvements in screening and prevention strategies. Through these measures, we aim to provide a clear understanding of program outcomes, highlight areas for improvement, and offer a cost-efficient approach to prevent hemoglobinopathy-related births.

## Highlights

- Effective screening for hemoglobinopathies prevents affected births. Success relies on counselor quality, testing accuracy, accessibility, timeliness, and demographic factors influencing participation and outcomes.
- Screening index measures the percentage of at-risk women successfully screened and informed, reflecting the program’s reach and timeliness.
- Prevention Index tracks the number of women enrolled per birth prevented, highlighting the program’s effectiveness in reducing affected births.
- These indicators help measure the effectiveness, cost-efficiency, and societal impact of screening programs, guiding improvements and resource allocation.

## Introduction

Targeted screening for hemoglobinopathies is vital for preventing affected births and avoiding psychological and financial hardship to involved families. To ensure effective long-term program monitoring, it’s essential to establish consistent, well-defined, timely, and reliable indicators. Ongoing efforts focus on identifying the optimal strategies for prevention, including the selection of testing technologies, operational structures, resource allocation, and program design. It is crucial to develop indicators that comprehensively measure the success of these interventions and also account for the impact of innate factors, such as variations in carrier rates among different populations.

*Screening pregnant women*: The first component focuses on providing access to carrier screening for the target population: pregnant women. A commonly used measure is the direct count of how many couples are offered screening. However, this indicator has limitations, as it does not account for testing failures or the lack of communication about results to the couples (1–3). By knowing the overall population size that requires screening, one can use this direct measure to estimate the reach of the program.

*Preventive component*: The second component involves enabling carrier families to access interventions that determine the disease status of the fetus, allowing for informed decision-making. This aspect is typically assessed through the reduction in birth rates, which reports the number of affected births before and after the implementation of prevention strategies. However, this indicator has a long lag period and relies heavily on universal newborn screening or a robust, centralized patient registry—both of which present significant challenges (4). This indicator has been widely utilized for many years (5–8).

To enhance monitoring efforts, we propose two additional indicators that may be widely applicable and could help build consensus on effective outcome measures.

## Key factors influencing screening and prevention success

To effectively understand screening outcomes, it’s essential to consider several influencing factors: the quality of counselors and coordinators, which enhances enrolment and patient satisfaction; the location of screening, as accessibility and infrastructure significantly affect participation rates; the effectiveness and accuracy of testing, ensuring that at-risk individuals are correctly identified; and the timeliness of screening processes, which can increase compliance. Finally, addressing technical limitations in testing are significant challenges as the debate continues on best technical strategy to screen individuals reliably. The existing indicator which represents enrolment does not include the impact of many of the above factors as it directly measures enrolment alone.

The role of access to safe options for pregnancy discontinuation is crucial, as is understanding societal issues and family preferences that can impact prevention. Innate factors also play a crucial role in influencing prevention outcomes. Geographic and demographic variations can significantly affect prevalence rates, while families with a history of hemoglobinopathies are more likely to participate in screening programs and seek prevention.

### Proposed indicators

To effectively measure success, we need indicators that provide a clear, comparative understanding across target populations while allowing adjustments for innate factors. We explore two new indicators which may be more relevant to the targeted prevention approach.

#### Screening index

Screeningindex(%)=Total number of successful timely screening and informedx100Size of target population

This metric indicates the percentage of at-risk women who were successfully screened. It measures not just enrolment, but also whether these women were informed of their carrier status. Timeliness is important, as screening must be completed promptly to allow time for partner and fetal screening, and for any necessary decisions regarding termination. This indicator excludes failures related to testing and communication of results to families. By using regional birth estimates or data on how many families accessed care during the designated screening period, this metric effectively assesses the outreach, enrollment, testing, and communication efforts within the screening process.

Since the screening program aims to raise awareness of carrier status, this indicator should account for women who were already aware of their carrier status. These women should be reported separately, as part of the overall Screening index.


Overallscreening index %=(Total number of successful timely screening and informed+Total number of women who already knew the carrier status) x 100Size of target population

#### Prevention index

$$PreventionIndex=\frac{\mathrm{Total number of women enrolled}}{\mathrm{Total births prevented}}$$

This measures the number of women enrolled for each birth prevented, a direct indicator of the eventual effectiveness of prevention efforts. The lower the number, the more successful the program. However, this indicator, when comparing different regions/programs, needs normalization to account for variations in prevalence.


NormalisedPreventionIndex=Total number of women enrolled x Overall carrier rateTotal births prevented x Carrier Rate

Overall carrier rate refers to carrier rate in the whole population. For example, the carrier rates specific to each state in India can be normalized to the national carrier rate while comparing the success of the programs across states.

The indicator still has a delay in terms of the gestational age within which enrollment can be converted into prevention, and it is considered final only after each at-risk family has passed the screening window. Furthermore, to account for families with a history of affected births, this indicator may be evaluated both with and without these families included.

This indicator has the additional advantage of having direct translation into the costs of prevention. By using the cost of 1 screening as the starting point one can convert this indicator into the cost for each prevention. Meaningful reflection of cost per prevention is a very powerful perspective into the need and success of preventive approaches.

Examples of scenarios where the indicators may be effective:

1. *Performance Comparison and Peer Benchmarking*: Evaluate the effectiveness of various centers, divisions, regions, and programs against one another.
2. *Assessment of New Approaches*: Compare innovative strategies, such as extending the program to Primary Health Centers (PHCs), with existing methods.
3. *Monitoring Coordinator Development*: Track the progress of coordinators by measuring their performance against established baseline expectations.
4. *Early Warning System for Underperformance*: Implement a system to identify centers or programs that are not meeting performance standards.

Examples of statements we will be able to make using these indicators:

1. In the province the screening index has increased from 4 to 9% just with the impact of our program.
2. Prevention index improved from 981 to 700 in the last 3 months indicating the improvements seen in the local processes.
3. The normalized prevention index for the high prevalence region was 700 while that for another low prevalence region was only 1,100.

## Conclusion

In summary, the success of a targeted screening program can be evaluated through two key measures: screening index and the normalized prevention index. This combined approach highlights immediate effectiveness and facilitates tailored interventions based on specific challenges identified during the screening process. Ultimately, it helps to more directly assess the true value delivered to society.

## Data Availability

The original contributions presented in the study are included in the article/supplementary material, further inquiries can be directed to the corresponding author.
